# A Multi-Center Structural Equation Modeling Approach to Investigate Interpersonal Violence Screening for Public Health Promotion

**DOI:** 10.3389/fpubh.2021.637222

**Published:** 2021-06-11

**Authors:** Lan Jiang, Melissa A. Sutherland, M. Katherine Hutchinson, Bing Si

**Affiliations:** ^1^Department of Systems Science and Industrial Engineering, Thomas J. Watson School of Engineering and Applied Science, Binghamton University, Binghamton, NY, United States; ^2^Decker School of Nursing, Binghamton University, Binghamton, NY, United States; ^3^College of Nursing, University of Rhode Island, Kingston, RI, United States

**Keywords:** structural equation modeling, multi-center data fusion, interpersonal violence screening, theory of planned behavior, healthcare providers

## Abstract

**Background:** Interpersonal violence is a significant public health issue. Routine health screening is a cost-effective strategy that may reduce harmful physical and mental consequences. However, existing research finds consistently low rates of violence screening offered by healthcare providers, e.g., nurses, nurse practitioners, physicians. There is a critical need for research that helps understand how providers' screening behaviors are impacted by individual-level and organizational-level factors to promote the uptake of routine screening for interpersonal violence. Two recent studies, i.e., The Health Care Providers study and Nurse Practitioners Violence Screening study, involved quantitative data collected to measure providers' screening behavior and multi-level factors impacting violence screening.

**Methods:** The current analysis includes a combination of multi-center data collected from The Health Care Providers and Nurse Practitioners Violence Screening studies, respectively. The total sample is 389 providers across the United States. The proposed research develops a system-level multi-center structural equation model framework to rigorously integrate data from the two studies and examine providers' screening behavior for interpersonal violence based upon Theory of Planned Behavior from a quantitative perspective.

**Results & Conclusions:** We successfully examine the efficacy of the Theory of Planned Behavior proposed by Ajzen to predict healthcare providers' screening behavior for interpersonal violence. Organizational factors, e.g., availability of policy for interpersonal violence screening, organizational priority given to violence screening relative to other priorities, and if providers within the health center are interested in improving care quality, were significantly associated with providers' screening behavior. The knowledge and insights generated from our study may facilitate the design and optimization of health professional training and practice environment, and lead to improved women's health and quality of care.

## Introduction

Healthcare screenings are vitally important and cost-effective prevention strategies to promote health for people of all ages. Preventative health screenings can identify disease or other health problems at an early stage so proactive treatments can be offered to lower the risk for the disease, inhibit its progression, or reduce other harmful health consequences (1, 2). There are various types of health screening tests available in today's healthcare systems. For instance, the American Diabetes Association recommends all adults be screened for diabetes starting at age 45 by evaluating the blood sugar levels through blood tests (3). Screening that uses mammography shows a great success in reducing breast cancer mortality rates among female patients (4). Osteoporosis is a disease that reduces the density and quality of bone and the United States Preventive Services Taskforce recommends screening for osteoporosis with bone measurement testing such as a dual energy X-ray absorptiometry scan to prevent osteoporotic fractures in women 65 years and older (5).

Among a variety of diseases and health issues, interpersonal violence, which includes intimate partner violence (IPV) and sexual violence (SV), has been increasingly recognized as a major public health problem. Intimate partner violence is defined as violence or aggression that occurs in a close relationship with intimate partners, who include current or former spouses and dating partners (6). It is estimated that 1 in 4 women will experience sexual violence, physical violence, and/or stalking by an intimate partner during their lifetime (6). Additionally, 20 percent of women are sexually assaulted in their lifetime and 1.3 to 5 million women experience interpersonal violence each year (6). According to a report from the World Health Organization, interpersonal violence results in acute and long-term effects on women's health, including injuries and death (7). Due to the prevalence and substantial health effects, screening for interpersonal violence has received a significant amount of attention as a secondary prevention strategy that may reduce the harmful consequences of violence and/or prevent the reoccurrence of further acts of violence (8). Different from prior examples of health screenings, i.e., diabetes screening, breast cancer screening, and osteoporotic screening, violence screening is primarily considered as a “patient care behavior” carried out by healthcare providers, such as nurses, nurse practitioners, and physicians, and is not dependent on timely and costly medical tests or examinations. Therefore, violence screening is a cost-effective prevention strategy for women's health promotion. However, existing studies report low rates of screening for interpersonal violence across various healthcare settings (9–11). Barriers to violence screening previously identified in literature include provider level factors (e.g., knowledge of interpersonal violence, provider comfort, self-efficacy) and clinic-level factors (e.g., protocols for screening) (12).

The prevalence and consequences of interpersonal violence make it imperative to further investigate healthcare providers' violence screening behaviors and identify potential factors that promote the uptake of routine screening. Findings may lead to improved women's health and quality of life. Studies have examined the potential influential factors associated with providers' interpersonal screening behavior (13–18), but many of these studies were qualitative. A few quantitative studies were completed but were limited by small and relatively homogeneous samples. Therefore, there is a need for large, more comprehensive data sets that involve larger samples of health care providers and their screening behaviors. Recognizing this need, the authors integrated two datasets from two separate studies related to healthcare providers' violence screening behaviors. The data were collected through the Health Care Providers (HCP) study (19–21) and the Nurse Practitioners Violence Screening (NPVS) study (22, 23). The scope of the HCP study was to study the violence screening behavior of providers at college health centers in a sample of 170 participants. The NPVS focused on healthcare providers in primary care with a final sample of 219 participants.

Both HCP and NPVS datasets were designed following the constructs of Theory of Planned Behavior (TPB) (24). TPB is a validated social psychological model to study human behavior and has been successfully applied to predict a wide range of human behaviors and intentions in various fields such as healthcare, public relations, and management (25–28). According to TPB, healthcare providers' screening behaviors should be determined by their behavioral intention to screen, which is further influenced by three constructs, i.e., the attitude toward behavior for violence screening, subjective norm of screening, and perceived behavioral control over screening. Following the Guidelines to Construct Questionnaires based on TPB (29), each construct can be further measured by a collection of questions carefully designed by researchers. Therefore, the two TPB-based survey datasets provide an opportunity to examine the relationship between providers' screening behavior and other TPB constructs. Furthermore, such complex relations among multiple constructs can be quantitively analyzed by structural equation modeling (SEM), which is a well-established statistical approach (30, 31).

Motivated by the recently available HCP and NPVS data, this paper proposes a cross-dataset multi-group SEM-based approach to integrate both datasets and investigate healthcare providers' violence screening behavior based on TPB. From the perspective of healthcare research, the inclusion of the wider population of participants in the study enables a more comprehensive understanding of providers' screening behaviors in various healthcare settings. From the perspective of data science research, combining multiple datasets could overcome challenges in data shortage and limited samples and increase the power of statistical models. There have been quite a few studies that focus on integrating/combining multiple datasets for a joint analysis (32–38). However, a number of challenges are posed when integrating multiple datasets for a comprehensive study. We take the integration of HCP and NPVS datasets as an example. First, simply pooling all observations from HCP and NPVS studies into a combined dataset is likely to increase the heterogeneity among participants, which results from potential differences in participant selection criteria adopted by different research teams. Secondly, because multi-center datasets are typically collected by different research teams at different time periods, it is common for each team to adopt their own measurements for the same variable of interests; another challenge when integrating multi-center datasets. A detailed discussion on the variable discrepany between HCP and NPVS studies can be found in sections Data Description and Building TPB Constructs by Integrating Variables from Two Datasets. An important consideration when combining multiple datasets is how to identify common data elements shared by all the datasets and integrate the common variables into the combined dataset.

The objective of this analysis is to examine healthcare providers' screening behaviors and related attitudes, beliefs, and practice environment to optimize providers' education, training, and workplace environment. A widely used and validated behavioral model, the Theory of Planned Behavior (24), was the theoretical model used to examine providers' screening behaviors and their attitudes, beliefs and practice environment characteristics *from a qualitative perspective*, while structural equation modeling was used to model and analyze the complex relationships specified by TPB *from a quantitative perspective*. The proposed study is a secondary analysis of two real-world datasets collected in HCP and NPVS studies. To address the two aforementioned challenges in multi-center data fusion, we propose a multi-group SEM that: (1) fits a separate model for each of two groups and offers the flexibility to enable joint estimation by imposing constraints to selected parameters between the two groups, instead of fitting a single SEM for the combined dataset; and (2) allows the same TPB constructs to be measured by similar but not identical items for each group of data to mitigate the potential discrepancy in cross-study variables. More details can be found in sections Building TPB Constructs by Integrating Variables from Two Datasets and A Multi-group Structural Equation Model (multi-group SEM). The proposed method contributes on/to both nursing research and data science research. From the data analysis perspective, combining multiple datasets could overcome challenges in data shortage and limited samples and increase the power of statistical models. For nursing research, a wider population of participants could be more representative of healthcare providers in general, and therefore help understand and examine the violence screening behavior of providers who work at various healthcare settings. The findings of this research can facilitate the design and optimization of education and training of healthcare providers to promote the uptake of routine screening for interpersonal violence, and may lead to improved patient outcomes and women's health.

## Methods

### Data Description

This study integrates two datasets collected from the Health Care Providers (HCP) study (19–21) and the Nurse Practitioners Violence Screening (NPVS) study (22, 23). Both studies were reviewed and approved by Boston College with the IRB Protocol Number 16.042.01 (for HCP) and IRB Protocol Number 18.067.01e (for NPVS). Online consent was obtained for both studies. The principal investigators of both studies designed the TPB-based survey questions and administered the questionnaire to healthcare providers across multiple healthcare centers in the U.S. In the questionnaire, violence screening behavior was measured by the screening percentage reported by each individual healthcare provider. Intention to perform screening was also measured. Providers' attitudes toward screening, subjective norm, and perceived behavior control were each measured by a collection of questions designed by two research groups based on literature review and investigators' clinical experience. The sample for this analysis includes 219 participants from the HCP study and 170 participants from the NPVS study, a total of 389 healthcare providers. Due to the limited space, a more detailed discussion on data collection procedure is skipped in this article and can be found in papers published by the HCP (19–21) and NPVS (22, 23) researchers.

Combining the datasets gives us the opportunity to perform this secondary data analysis, investigate violence screening behavior using the TPB on a relatively heterogeneous population, and enhances the power of the statistical analysis. However, integrating multi-center datasets typically poses a challenge in collecting common data elements across different datasets and studies. In the following section, more details are discussed in regard to identifying common variables/survey questions shared by both datasets and leveraging similar but not identical variables (questions) in this cross-dataset analysis.

The aim of the analysis was to use the data from two independent datasets to examine healthcare providers' interpersonal violence screening behavior from the perspective of TPB, and therefore only questions that describe providers' screening behavior, intention, and the other three TPB constructs, which include their attitudes toward screening, subjective norm, and perceived behavioral control, are included in the combined datasets. A detailed comparison of the list of questions included from both HCP and NPVS datasets is shown in Table 1, of which two components are treated as observed and the other three are latent and need to be measured by a few items. Specifically, the two observed components are “screening behavior” and “screening intention,” which are defined as “ the percentage of female patients (college students) you screen for IPV/SV” and “how likely you would screen all female patients (college students) for IPV/SV,” respectively, while “attitude toward behavior,” “subjective norm,” and “perceived behavioral control” are latent variables. Note that all items used to measure latent variables are ordinal. For instance, one of the items to measure the “attitude toward behavior” is “In your opinion, is it a bad or good idea to routinely screen for IPV with every female patient?” with options that include “very bad idea,” “bad idea,” “neutral,” “good idea,” and “very good idea,” which results in a five-point Likert score.

**Table 1:** Summary of HCP and NPVS studies' questions included in TPB analyses.

		**Questions**	
Screening Behavior	HCP	Of the female college students who you saw at the college health center during the past 2 months, approximately how many (what percentage) did you screen IPV?	
	NPVS	Of the female patients who you saw during the past 3 months, approximately what percentage did you screen for IPV and SV?	
Screening Intention	HCP	How unlikely or likely is it that you will routinely screen all female students for IPV and SV during the next 2 months?	
	NPVS	How likely is it that you would routinely screen all female patients for IPV and SV?	
Attitude Toward Behavior	HCP	In your opinion, is it a bad or good idea to routinely screen for IPV with every female student who visits the college health center?	
		In your opinion, is it a bad or good idea to routinely screen for SV with every female student who visits the college health center?	
	NPVS	In your opinion, is it a bad or good idea to screen for IPV with every female patient who visits your health care setting?	
		In your opinion, is it a bad or good idea to screen for SV with every female patient who visits your health care setting?	
Subjective Norm	HCP	Normative beliefs	*Please indicate how strongly you disagree or agree with each of the following statements about your co-worker's attitudes*.
			(a.1) Would your colleagues disapprove or approve if you routinely screened female patients for IPV/SV in your college health center?
			(b.1) Would the director of the college health center disapprove or approve if your routinely screened female patients for IPV/SV?
		Motivation to comply	(c.1) How important is it to you that your colleagues in the college health center approve of what you are doing?
			(d.1) How important is it to you that the director of the college health center approves of what you are doing?
	NPVS	Normative beliefs	(a.2) Nurses in my workplace would approve of offering IPV and SV screening to all female patients.
			(b.2) Nurse Practitioners (NPs) in my workplace would approve of offering IPV and SV screening to all female patients
			(c.2) Physicians in my workplace would approve of offering IPV and SV screening to all female patients.
			(d.2) The health center director in my workplace would approve of offering IPV and SV screening to all female patients
		Motivation to comply	(e.2) How important is it to you that the Nurses you work with approve of what you are doing?
			(f.2) How important is it to you that the Nurse Practitioners (NPs) you work with approve of what you are doing?
			(g.2) How important is it to you that the Physicians you work with approve of what you are doing?
			(h.2) How important is it to you that the health center director approves of what you are doing?
Perceived Behavioral Control	HCP	I am confident that I could screen for IPV and SV, during the next 2 months.	
		I am confident that I could perform a danger assessment, during the next 2 months.	
		I am confident that I could discuss safety planning, during the next 2 months.	
		I am confident that I could refer students who screen positive for follow-up and counseling, during the next 2 months.	
	NPVS	I am confident that I could screen for IPV and SV.	
		I am confident that I could perform a danger assessment with patients.	
		I am confident that I could discuss safety planning with patients.	
		I am confident that I could refer patients who screen positive for follow-up and counseling.	

### Building TPB Constructs by Integrating Variables From Two Datasets

The Theory of Planned Behavior is a social psychological model of human behavior developed by Ajzen (24). As aforementioned, TPB predicts providers' screening behavior by their intention to screen, while their intentions are associated with three constructs, i.e., their attitudes toward screening, subjective norm, and perceived behavioral control toward screening. Table 1 shows how each construct in TPB is measured by a collection of questions. Besides the TPB constructs, items focused on organizational factors were collected as part of the study, that could potentailly impact the healthcare provider's behavior as well.

Two major differences between the dataset (HCP and NPVS) should be noted. The HCP study focused on investigating interpersonal violence screening for college women, and therefore recruited healthcare providers from college health centers. In the NPVS study, all participates worked in primary care and were recruited through the mailing list of American Association of Nurse Practitioners (https://www.aanp.org). Therefore, a difference between the two sets of survey questions is that NPVS focused violence screening for *all female patients* while HCP focused on screening behavior for *college female students*. Another difference was that the recall of behavior and intention were different for HCP and NPVS studies. For example, HCP dataset asked about screening percentages for the past 2 *months*, while NPVS dataset measured “screening behavior” that used providers' self-reported screening percentages for the past 3 *month*s. The combination of two datasets creates a relatively heterogeneous population of healthcare providers with more subjects for the investigation of interpersonal violence screening behavior under a variety of clinical care settings.

To provide a composite measure for each construct, Guidelines on Constructing TPB Questionnaire (29) has different options for questions' design. Depending on which set of questions has been chosen to be measured for the construct, the guideline could make a specific recommendation to produce an effective composite measure from these questions. Therefore, the flexibility of the guidelines in producing composite scores allows us to first identify a set of common questions shared by both datasets and then choose the measurement that is most suitable to produce/compute a composite score based on the common questions set we identify from HCP and NPVS datasets. More details of each of the TPB constructs along with a list of organizational factors are described as follows:

#### Screening Behavior and Intention

Violence screening behavior was measured by providers' self-reported screening percentage on a scale from 0 to 100%. The screening intention was evaluated by asking providers “how likely is it that you would routinely screen all female patients/students for IPV and SV,” rated on a five-point Likert scale from “Very Unlikely” to “Very Likely.” It is noteworthy that the HCP study used a 2-month recall of screening behavior and intention and NPSV used a 3-month recall.

#### Attitude Toward Behavior

As shown in Table 1, attitude toward screening behavior was directly measured by two questions, i.e., “in your opinion, is it a bad or good idea to screen for IPV with every female patient/student who visits your healthcare setting?” and “In your opinion, is it a bad or good idea to screen for SV with every female patient/student who visits your healthcare setting?” According to the guidelines (29), the two items were considered as direct measurements of screening attitudes to evaluate the overall attitude toward screening.

#### Subjective Norm

According to Guidelines on Constructing TPB Questionnaire (29), both HCP and NPVS adopt indirect measurement questions to evaluate the subjective norm. That is, the subjective norm is evaluated indirectly by a belief-based measurement, which is obtained by computing the product between “normative beliefs” and “motivation to comply.” In Table 1, “normative beliefs” are measured by the questions such as “would any *individual or group* disapprove or approve if you routinely screened female students/patients for IPV/SV in your healthcare center?,” while the “motivation to comply” is measured by the corresponding question of “How important is it to you that this *individual or group* in the healthcare center approve of what you are doing?” For instance, the NPVS study collected providers' normative beliefs and motivation to comply with the beliefs about nurses, Nurse Practitioners, physicians, and health center directors, which are listed as questions (a.2), (b.2), (c.2) and (d.2) in Table 1, while HCP grouped the four roles into two categories that consist of colleagues who work with you and your health center directors. This resulted in two questions (a.1) and (b.1) under normative beliefs. By combining the questions for “normative beliefs” and “motivation to comply,” the overall subjective norm for HCP subjects is measured using two items, i.e., (a.1) × (c.1) and (b.1) × (d.1), while the subjective norm for NPVS subjects is measured using four items, i.e., (a.2) × (e.2) , (b.2) × (f.2) , (c.2) × (g.2), and (d.2) × (h.2).

#### Perceived Behavioral Control

Providers' perceived behavioral control toward screening was directly measured by evaluating how much the provider has control over interpersonal violence screening-related behavior. For example, “I am confident that I could screen for IPV and SV” is a question that measures how much control the provider has over screening. In Table 1 under “Perceived Behavioral Control,” there are four items that measure how confident the providers are at performing different screening-related assessments and their perceived behavioral control.

#### Organizational Factors

Recognizing that provider behavior is influenced by the organizational factors, both HCP and NPVS studies collected data on providers' perceptions of organizational characteristics in their practice settings [56]. This study included five organizational items: (1) Does your health center routinely screen all patients for IPV and SV? (2) How strongly do you agree that “IPV and SV screening is a top priority in this health center?” (3) How strongly do you agree that “There is a big push to screen all female patients for IPV and SV?” (4) How strongly do you agree that “People here put a lot of effort to ensure adherence to IPV and SV screening guidelines;” (5) How strongly do you agree that “Providers are very interested in improving care quality.” The five organizational items were self-reported by providers based on their individual perceptions of the health settings where they practiced.

### A Multi-Group Structural Equation Model

SEM is a well-established statistical approach to represent, estimate, and test complex relations among variables, among which the relationship is usually specified according to domain knowledge (30, 31). As an extension of the traditional SEM, the mutli-group SEM examines structural relations in two or or more groups, in which the structural relations can be investigated across groups to determine whether underlying constructs differ or any particular items perform poorly in one group or another. Both SEM and multi-group SEM have been implemented in a number of statistical software. In particular, this study borrows strength from a build-in package “lavaan” (39) in an open-source statistical software R (40) for multi-group SEM esimtation. Aided by R, users only need to specifiy the relations among constructs for each group and the R software automatically provides the model estimation.

**Figure 1:**
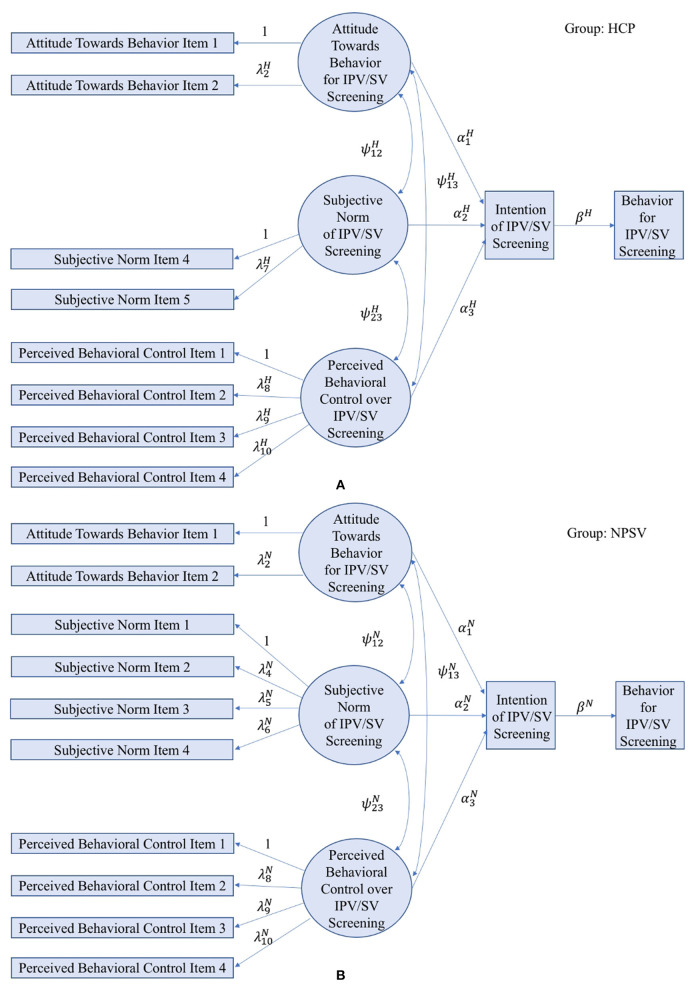
Model specification for the multi-group SEM in the HCP study **(A)** and the NPVS study **(B)**.

Next, we introduce the multi-group SEM following a five-step approach (41, 42) that consists of model identification, specification, estimation, testing, and re-specification. The model identification and specification for the multi-group SEM is based on the TPB theory, which specified a theoretical framework to describe the relationship between attitude toward behavior, subjective norm, and perceived behavioral control and their impact on screening intentions and behaviors. A graphical representation of the SEM specification can be found in Figure 1, in which Figure 1A presents the specification for HCP dataset and Figure 1B presents the specification for NPVS dataset. We take Figure 1A as an example. Observed variables are indicated by rectangles and latent variables are indicated by ellipses. Parameters of SEM include regression coefficients, covariance, and variance. The regression coefficients are represented along the single-headed arrows. The double-headed, curved arrows between two variables indicates the covariance, a non-causal association relationship. The variance can be represented by the double-headed, curved arrows, both heads of which point to the same variables. Note that variances are not shown in this paper's figures to simplify the SEM diagrams but are available upon request. As discussed in section Building TPB Constructs by Integrating Variables from Two Datasets, the two datasets use different items to measure the construct of “subjective norm;” therefore a multi-group SEM is adopted to integrate the two groups of data while allowing for similar but not identical items to be used to measure the same construct. Specifically, the structural models have the same forms for two datasets based on the TPB theory, while the measurement models are slightly different depending on which individual items are used to measure “subjective norm.” Considering the total estimable parameters and total number of parameters to be estimated, a degree of freedom is calculated to determine if the proposed SEM is identifiable or not. For identifiable models, different parameter estimation approaches are available for structural equation modeling, which depends on the type of variables. In this study, the robust maximum likelihood estimation is selected due to its reliable performance in handling ordinal variables (43). The overall model fitness is evaluated by multiple criteria such as likelihood ratio test (LRT), comparative fit index (CFI), Tucker-Lewis Index (TLI), and Root Mean Square Error of Approximation (RMSEA) (44, 45).

In the initial model specification, multi-group SEM does not specify any constraints on the parameters between the two groups, whose results are introduced in section An Unconstrained Multi-group SEM Applied to TPB-based Violence Screening Behavior. Later, this study explores imposing constraints to ensure equal coefficients in the structural models to re-specify the SEM for comparison. The re-specified model can be found in section A Constrained Multi-group SEM Applied to TPB-based Violence Screening Behavior. Both sections specify the structural models and measurement models based on the traditional TPB theory without organizational influence. In recent years, organizational factors have shown increasing influences on providers' behaviors but their relationship with the TPB constructs remains unknown. To explore organizational expansion of the TPB theory, section Organizational Expansion of TPB-based Violence Screening Behavior investigates organizational factors' relationship with the TPB constructs through model specification and re-specification. The optimal model is recommended based on the goodness-of-fit, which facilitates the understanding of how organizational characteristics are related to providers' attitudes, beliefs, intentions, and behaviors for interpersonal violence screening for the optimization of providers' training, education, and workplace environment.

## Results

The survey data from HCP and NPVS were first cleaned and then coded or reversely coded as necessary. Before combining the data of the two datasets, Chi-squared tests were used to compare demographics of participants from two different studies, which indicated no significant difference between the two groups of participants in age (*p*-value = 0.06), gender (*p*-value = 0.08), and race (*p*-value = 0.93). No statistically significant demographics difference was noted between the two groups; this provides support to justify the combination of the two data sources.

Prior to the structural equation modeling, the HCP and NPVS data are tested for validity, consistency and reliability. First, to examine if the data is suitable for factor analysis, Bartlett's Tests of Sphericity (46) and Kaiser-Meyer-Olkin Tests (47) are performed on two groups of data, respectively. Based on Bartlett's Tests of Sphericity, there is sufficient significant correlation in the two datasets for factor analysis with *p*-value <0.001. The Kaiser-Meyer-Olkin measures are 0.78 and 0.72 for HCP data and NPVS data, respectively, which indicate a strong correlation between factors. Moreover, Confirmatory Factor Analysis is conducted to establish the construct validity by testing the relationships between latent constructs and their individual items, which reports consistent results for both datasets. The likelihood ratio tests result in *p*-values <0.001. CFIs and TLIs are both higher than 0.95, while RMSEAs are <0.08 (44, 45). The loadings, i.e., coefficients of the measurement models, are all statistically significant with *p*-values <0.001. The results indicate a good fit between the datasets and the measurement model, thus establishing the construct validity.

The reliability and internal consistency among items within the same construct are examined by Cronbach's alpha. As shown in Table 2, Cronbach's alphas are calculated for HCP, NPVS, and the combined dataset. Cronbach's alphas >0.8 show strong internal consistency within “attitude toward behavior,” “subjective norm,” and “perceived behavior control,” except for “subjective norm” in HCP dataset, which has an alpha of 0.687. However, by combining HCP data with NPVS data, the lower consistency is mediated, and thus the combined dataset shows strong internal consistency for each of the three constructs. Specifically, as shown in Table 2, the construct that represents attitude toward behavior is directly measured by providers' attitude toward screening for IPV (item 1) and SV (item 2), which has a high Cronbach's alpha of 0.934. The construct of subjective norm is indirectly assessed by the products of normative beliefs and motivation to comply with the beliefs, which are labeled as items 11–14 in the table. The subjective norm construct in the combined dataset has a strong internal consistency with a Cronbach's alpha of 0.814. The construct for perceived behavioral control is directly evaluated from items 15–18, which measure providers' capability to preform interpersonal violence screening-related assessments.

**Table 2:** Cronbach's alphas to examine internal consistency of each TPB construct.

**Construct**	**Item**	**HCP**	**NPVS**	**Combined dataset**
Attitude Toward Behavior	Item 1: screen for IPV Item 2: screen for SV	0.958	0.907	0.934
Subjective Norm^*^	Item 3: nurses' approval Item 4: NPs' approval Item 5: physicians' approval Item 6: director's approval	0.687	0.910	0.814
	Item 7: compliance with nurses Item 8: compliance with NPs Item 9: compliance with physicians Item 10: compliance with director			
	Item 11: item 3 × item 7 Item 12: item 4 × item 8 Item 13: item 5 × item 9 Item 14: item 6 × item 10			
Perceived Behavioral Control	Item 15: capability to screen for IPV and SV Item 16: capability to perform a danger assessment Item 17: capability to discuss safety planning Item 18: capability to refer patients for counseling	0.894	0.838	0.874

* *HCP data has fewer items under subjective norm because it groups “nurses,” “NPs,” “physicians” into “colleagues” and collects only subjective norm related to “colleagues” and “director.”*

An open-source statistical software R (40) was used for data processing and model development. Specifically, we take advantage of the existing R package “psy” (48) to examine internal consistency and reliability, R package “parameters” (49) to evaluate validity, and R package “lavaan” (39) for statistical modeling of multi-group SEMs.

### An Unconstrained Multi-Group SEM Applied to TPB-Based Violence Screening Behavior

**Figure 2:**
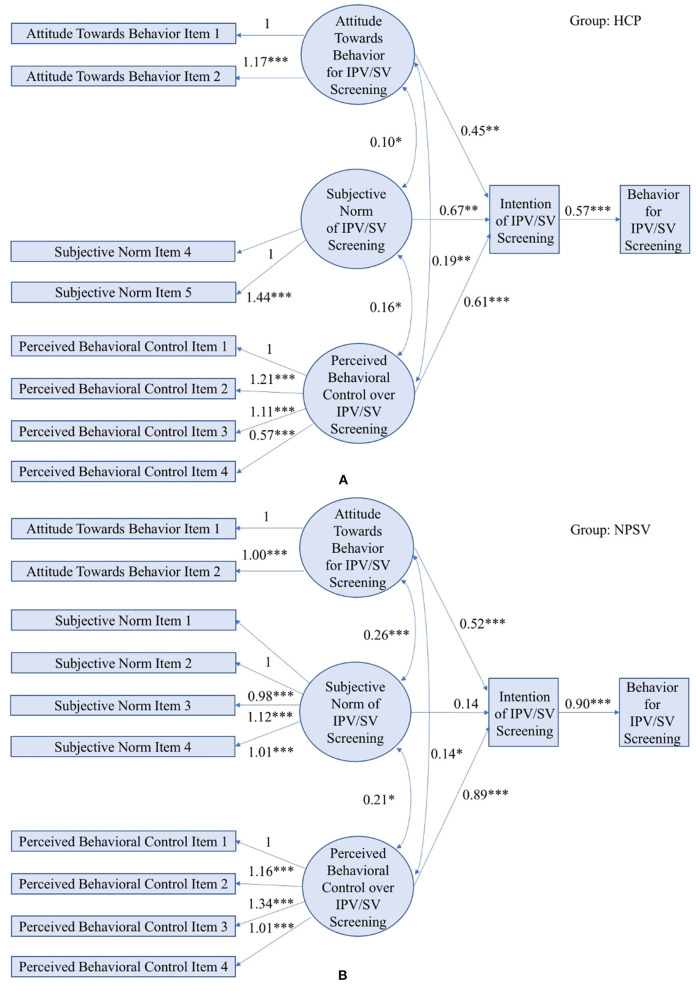
An unconstrained multi-group SEM applied to TPB in the HCP study **(A)** and the NPVS study **(B)**. (Model fit indices: LRT *p*-value < 0.001, CFI = 0.96, TLI = 0.94, RMSEA = 0.074; *** indicates *p*-value < 0.001, ** indicates *p*-value < 0.01, * indicates *p*-value < 0.05).

A multi-group Structural Equation Model was applied to the combined dataset to model, estimate, and test the relationship among interpersonal violence screening behavior, intentions, providers' attitudes toward behavior, subjective norm, and perceived behavior control. Structural models are the same for the two groups, while one of the measurement models, i.e., the model for measuring “subjective norm,” includes different individual items for different groups. No constraints on the parameters between the two groups are considered. Note that fitting an unconstrained two-group SEM is equivalent to fitting two SEMs for the two groups separately, since parameters for both groups are freely estimated without any constraints imposed. From Figure 2, we can see the total numbers of observed variables for the two groups are 10 and 12, respectively, and therefore the total number of estimable parameters is $\frac{10\times \left(10+1\right)}{2}+\frac{12\times \left(12+1\right)}{2}\;=\;133$. Considering the number of parameters to be estimated in Figure 2, the degree of freedom for the multi-group SEM is 79. Due to the presence of ordinal variables, a robust maximum likelihood estimation method is used for model estimation (43). The good fitness of the model demonstrates the efficacy of the Theory of Planned Behavior in understanding healthcare providers' violence screening behavior, with CFI = 0.96, TLI = 0.94, and RMSEA = 0.074.

The path coefficients are reported in Table 3, which consists of both unstandardized coefficients (USCs) and standardized coefficients (SCs). USCs are the coefficients reported in the SEM diagram as shown in Figure 2. In path analysis, USCs are typically rescaled to SCs to place the coefficients in units of standard deviations of the mean and allow coefficients belonging to different paths to be compared. That is, the expected impact of one standard deviation difference in one variable can be compared to one standard deviation difference in another variable. As a result, we can observe from Table 3 that providers' perceived behavioral control is a stronger predictor to intention, compared with their attitude and subjective norm.

**Table 3:** Path coefficients of the unconstrained multi-group SEM.

**Paths of structural models (Unconstrained)**	USC^*^		SC^**^		***P*****-value**	
	**HCP**	**NPSV**	**HCP**	**NPSV**	**HCP**	**NPSV**
Attitude toward behavior & intention	0.45	0.52	0.23	0.29	0.001	<0.001
Subjective norm & intention	0.67	0.14	0.29	0.10	0.001	0.255
Perceived behavioral control & intention	0.61	0.89	0.40	0.51	<0.001	<0.001
Intention & screening behavior	0.57	0.90	0.28	0.43	<0.001	<0.001

* *USC, unstandardized coefficient;*

** *SC, standardized coefficient*.

Figure 2 describes the quantitative relations estimated by the multi-group SEM. Except one, every other arrow that links two constructs is labeled with a *p*-value <0.05, which indicates a statistically significant association between the two constructs. Comparing the two groups, provider's intentions of screening are largely affected by their attitudes and perceived behavioral control rather than the subjective norms of screening. For both datasets, the coefficients for attitude toward behavior and perceived behavioral control constructs are significant, while the coefficient for subjective norm has a relatively smaller magnitude and is found to significant only in the HCP data. One of the possible reasons is that the sample size of NPSV data is smaller than that of HCP data, in which small effects, e.g., influence of subjective norms on intentions, is characterized by small coefficients that are difficult to be identified in analysis. As a result, the next section explores a constrained multi-group SEM with joint learning of structural models to leverage common variables shared by both HCP data and NPSV data to improve the statistical analysis with an increased sample size. Another possible reason may be due to the variations in violence screening behaviors among healthcare providers who work in different healthcare settings, which is a typical drawback for multi-center data fusion and analysis and is further elaborated in the discussion section.

Note that as a conventional statistical model, SEM typically cannot be used to identify any causal relationships between constructs/variables. However, the significant correlations discovered by SEM could partially support and verify the causations among constructs proposed by TPB from a quantitative perspective.

### A Constrained Multi-Group SEM Applied to TPB-Based Violence Screening Behavior

Section An Unconstrained Multi-group SEM Applied to TPB-based Violence Screening Behavior discusses a free multi-group SEM model to analyze HCP data and NPVS data with no constraints imposed on the coefficients. To explore the similarity in relationships between the two groups, this section presents a constrained multi-group SEM by assuming the same coefficients in the structural models, i.e., $\alpha_{1}^{H}=\alpha_{1}^{N}$, $\alpha_{2}^{H}=\alpha_{2}^{N}$, $\alpha_{3}^{H}=\alpha_{3}^{N}$, and *β*^*H*^ = *β*^*N*^. As a result, the number of parameters to be estimated is reduced by four, and the degree of freedom is increased from 79 to 83.

**Figure 3:**
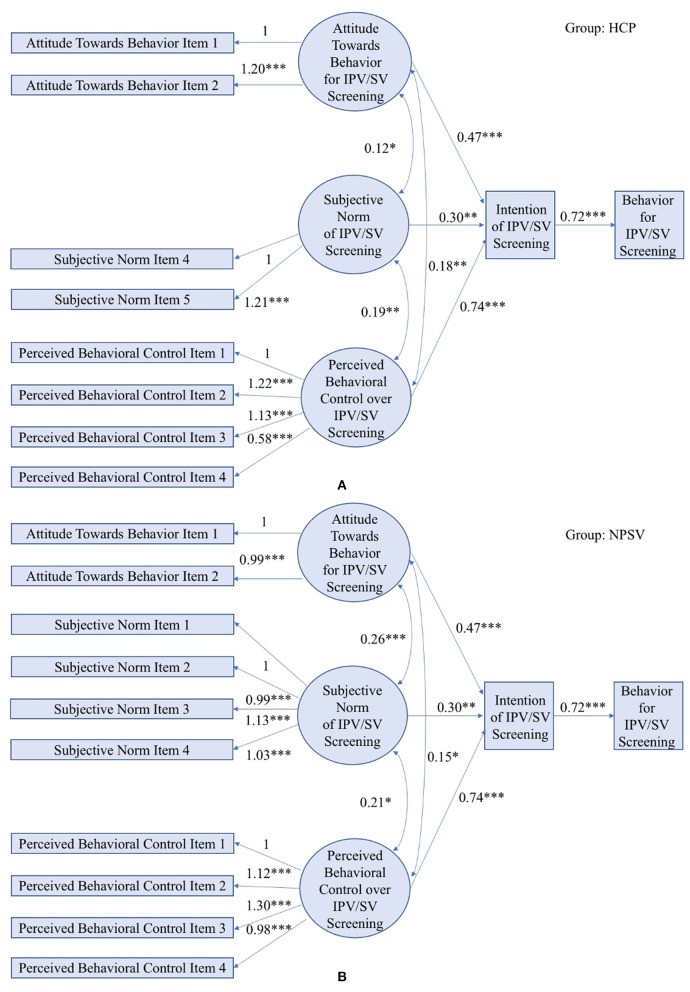
A constrained multi-group SEM applied to TPB in the HCP study **(A)** and the NPVS study **(B)**. (Model fit indices: LRT *p*-value < 0.001, CFI = 0.96, TLI = 0.94, RMSEA = 0.075; *** indicates *p*-value < 0.001, ** indicates *p*-value < 0.01, * indicates *p*-value < 0.05).

Figure 3 describes the quantitative relations estimated by the multi-group SEM. The coefficients in structural models are equivalent between two groups and are all significant in terms of *p*-values. The goodness-of-fit testing indicates that the data fits well in the constrained multi-group SEM, with CFI = 0.96, TLI = 0.94, and RMSEA = 0.075. The estimated SEM diagram is shown in Figure 3. All the hypothesized paths in both datasets are found to be significant in terms of *P*-values as shown in Table 4, in which USCs are the same for the two groups due to the imposed constraints, but SCs are resulted from standardization of USCs based on group-specific standard deviations and thus different between the two groups of data. Path coefficients indicate providers' perceived behavioral control remains to be a stronger predictor to intention in the constrained model, compared with their attitude and subjective norm. From the perspective of model fitness and model estimation, the consistent results between the constrained model and unconstrained model justify the use of the multi-group modeling approach, which leverages cross-dataset information as well as mitigates the slight discrepancy that resulted from data collection. From the perspective of nursing research, the constrained model indicates the efficacy and stability of using TPB to model healthcare providers' behaviors toward violence screenings across various healthcare settings.

**Table 4:** Path coefficients of the constrained multi-group SEM.

**Paths of structural models (Constrained)**	USC^*^	SC^**^ (HCP)	SC^**^ **(NPSV)**	***P*-value**
Attitude toward behavior & intention	0.47	0.24	0.26	<0.001
Subjective norm & intention	0.30	0.14	0.21	0.004
Perceived behavioral control & intention	0.74	0.47	0.44	<0.001
Intention & screening behavior	0.72	0.35	0.35	<0.001

* *USC, unstandardized coefficient;*

** *SC, standardized coefficient*.

### Organizational Expansion of TPB-Based Violence Screening Behavior

Beyond the behavioral study of individual healthcare providers, there is a growing consensus that provider behavior is influenced by organizational characteristics of the healthcare settings where they work (50). Existing literature proposed a list of potential organizational factors that impact the healthcare provider's behavior, among which we identify three common factors collected by both HCP and NPVS to further verify the organizational expansion of TPB. Based on the literature review and researchers' experiences, the organizational construct was measured using five items related to availability of policy to screen all female patients at the healthcare center, organizational priorities given to violence screening relative to other priorities, and if providers within the health center are interested in improving care quality. Construct validity was examined based on confirmatory factor analysis with CFI = 0.98, TLT = 0.97, and RMSEA = 0.07.

**Figure 4:**
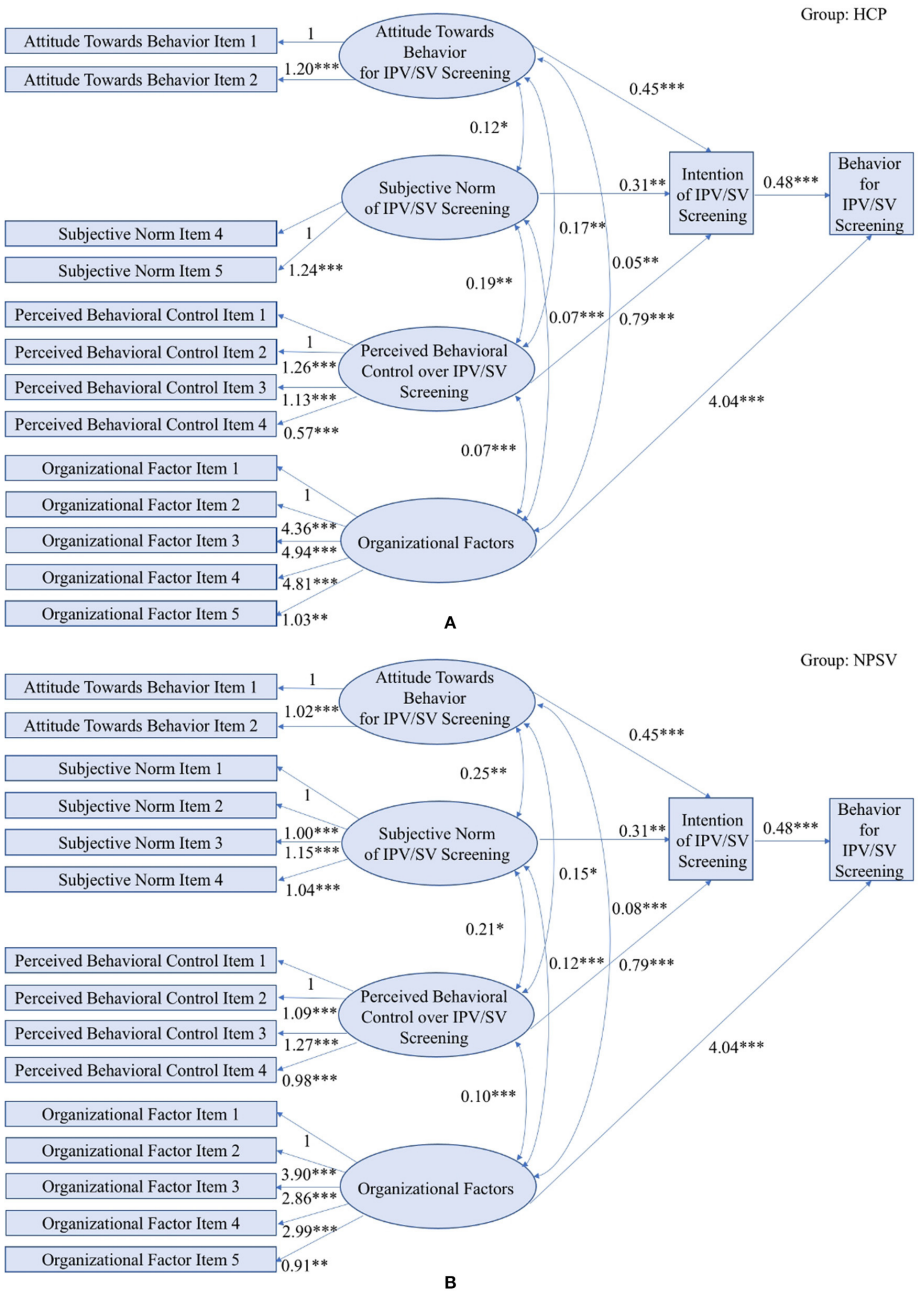
Estimated SEM applied to Organizational Expansion of TPB in the HCP study **(A)** and the NPVS study **(B)**. (Model fit indices: LRT *p*-value < 0.001, CFI = 0.93, TLI = 0.92, RMSEA = 0.074; *** indicates *p*-value < 0.001, ** indicates *p*-value < 0.01, * indicates *p*-value < 0.05).

To determine how TPB constructs are impacted by the organizational factors, we re-specify the multi-group SEM by adding the direct effect from the organizational factors to different TPB constructs. The final model is selected based on the model fitness, which is shown in Figure 4. The model fitness indices indicate a good fit between the HCP data and NPVS data and the multi-group SEM with LRT's *p*-value < 0.001, CFI = 0.93, TLT = 0.92, and RMSEA = 0.074. Path coefficients are summarized in Table 5, in which all hypothesize paths are shown to be significant with *p*-values <0.001. Comparing standardized coefficients of all three constructs impacting the intention, one standard deivation difference in the provider's perceived behavioral control leads to the greatest impact on his or her intention to screen for violence with the coefficients being 0.5 and 0.47 for HCP and NPVS studies, respectively. In addition to three newly added arrows, *p*-values of other arrows remain significantly small, which indicates the validity of adopting organizational expansion of TPB to predict providers' behavior for interpersonal violence screening.

**Table 5:** Path coefficients of the multi-group SEM with organizational expansion.

**Paths of structural models (Constrained model)**	USC^*^	SC^**^ **(HCP)**	SC^**^ **(NPSV)**	***P*-value**
Attitude toward behavior & intention	0.45	0.23	0.25	<0.001
Subjective norm & intention	0.31	0.15	0.21	0.004
Perceived behavioral control & intention	0.79	0.50	0.47	<0.001
Intention & screening behavior	0.48	0.22	0.24	<0.001
Organizational factors & screening behavior	4.04	0.28	0.36	<0.001

* *USC, unstandardized coefficient;*

** *SC, standardized coefficient*.

## Conclusions

This paper developed a multi-group Structural Equation Model integrated with Theory of Planned Behavior (29) to understand healthcare providers' interpersonal violence screening behavior and its relationship to providers' screening intention, attitude toward screening behavior, subjective norm, and perceived behavioral control. Two questionnaire-based studies, HCP and NPVS, are integrated into a combined dataset by identifying common or similar questions shared by both studies. The combined multicenter dataset results in a relatively larger and heterogeneous population in this study. Based upon Guidelines on Constructing TPB-based Questionnaires (29), each TPB construct that consists of a collection of survey questions is examined with Cronbach's alpha and showed a strong internal consistency with Cronbach's alpha >81%. To leverage information from both datasets and mitigate their discrepancy, a multi-group structural equation model was applied to the two datasets to investigate the TPB (24) constructs from a quantitative perspective. The good fitness of the structural equation model validated the utility of adopting the Theory of Planned Behavior (24) to study healthcare providers' behavior for violence screening. Furthermore, the organizational expansion of TPB was assessed by examining how TPB constructs were related to organizational items. These items included the availability of policy to screen all female patients at the healthcare center, the organizational priority given to violence screening relative to other priorities, and provider interest in improving care quality within the health center.

In the field of nursing research, this study is the first to examine the efficacy of the Theory of Planned Behavior (24) with and without organizational expansion, and to find adequate model fit and significant hypothesized paths. This study supports that providers' IPV and SV screening behavior is determined by their screening intentions, attitude toward screening, subjective norms, perceived behavioral control, as well as organizational factors. The knowledge discovered can be used to inform further interventions, such as educating and training healthcare providers and implementing screening policy at healthcare centers. Achieving this goal is important to today's healthcare system because routine screening is considered as a cost-effective strategy to help identify victims. The uptake of routine screening may reduce the harmful consequences of interpersonal violence and prevent the reoccurrence of further acts of violence, which leads to improved women's health and quality of life.

## Discussions

This analysis demonstrates the efficacy of TPB in predicting healthcare providers' screening behaviors for interpersonal violence. Based on the SEM results, providers' behaviors are impacted by their intentions, which in turn are affected by their attitudes toward screening, subjective norms, and perceived behavior controls. The findings are consistent with existing literatures. The efficacy of TPB in predicting human behaviors from their intentions, attitudes, and beliefs has been demonstrated in various behavioral studies in healthcare. Guo et al. (51) found healthcare administrators' intentions to use evidence-based management were significantly associated with their attitudes and perceived behavioral control. In another study, perceived behavioral control has been identified as the strongest predictor for healthcare providers' sexual counseling behaviors in people with epilepsy (52). Lin et al. (53) adopted TPB to study the help-seeking behaviors for sexual problems among women, which reported self-stigma and perceived behavioral control had significant effects on help-seeking behaviors (23).

Furthermore, this study identified a few organizational variables as additional factors that impact providers' behaviors for violence screening, which are comparable with existing studies in healthcare practices. In an NIH-funded pilot study on violence screening among college female students, the availability of policy to screen all female students was also shown to be positively associated with providers' screening percentages for interpersonal violence (54). In addition, our study reported that organizational priorities focused on violence screening relative to other priorities and health center providers interested in improving care quality were factors that impact providers' violence screening behaviors. Similarly, Solberg (50) proposed a conceptual framework on identifying facilitators to improve medical practices, in which higher organizational priorities and a higher degree of involvement and engagement by personnel at all levels were shown to be positively associated with quality improvement in healthcare practices.

There are several limitations of this analysis that are common challenges inherent in a secondary multicenter cross-dataset analysis: (1) The analysis results are built upon the 389 providers collected from previous studies and therefore may suffer from bias of participant recruitment; (2) method biases that could arise from a variety of sources, e.g., the content of specific items, scale types, and response formats, may result in measurement errors and need to be examined in future studies; (3) the TPB constructs included in the analysis are produced from common questions shared by both datasets, and there are other approaches listed in the Guideline of TPB-based Survey, which could be used for question design and composite score computation; (4) the sample size of the HCP and NPVS data is sufficient for structural equation modeling, but more data at an individual level and organizational level are needed to examine the presence of ecological fallacy in providers' screening behaviors; and (5) the organizational factors used to test for organizational expansion of TPB are limited to common factors shared by both datasets, but future studies should examine additional factors that could potentially impact the behavior of providers who work at different healthcare centers.

## Data Availability Statement

The data will not be deposited to any public repository but will be available upon request from the corresponding author. Requests for data access would be subject to review by researchers from the Health Care Providers (HCP) research steering group and by researchers from the Nurse Practitioners Violence Screening (NPVS) research steering group to ensure congruence with equity research goals. Requests to access the datasets should be directed to Bing Si ( bsi@binghamton.edu).

## Ethics Statement

This paper analyzed data collected from human subjects in the HCP and NPVS studies. Both studies were reviewed and approved by the Institutional Review Board at Boston College with the IRB Protocol Number 16.042.01 (for HCP) and IRB Protocol Number 18.067.01e (for NPVS). Online consent was obtained for both studies. The patients/participants provided their written informed consent to participate in this study.

## Author Contributions

All authors listed have made a substantial, direct and intellectual contribution to the work, and approved it for publication.

## Conflict of Interest

The authors declare that the research was conducted in the absence of any commercial or financial relationships that could be construed as a potential conflict of interest.
